# EXTREME HEAT EXPOSURE AND EPIGENETIC AGING ACCELERATION AMONG OLDER ADULTS IN THE US

**DOI:** 10.1093/geroni/igae098.1253

**Published:** 2024-12-31

**Authors:** Eunyoung Choi, Jennifer Ailshire

**Affiliations:** University of Southern California, Los Angeles, California, United States; University of Southern California, Los Angeles, California, United States

## Abstract

Extreme heat exposure is a major risk factor for cardiovascular diseases and mortality. However, knowledge about the biological ramifications of extreme heat exposure is limited, particularly among older adults who face elevated vulnerability. This study examines the association between heat exposure and epigenetic aging, a key molecular marker of biological aging based on DNA methylation (DNAm) levels throughout the genome. We used data on 3,414 US adults aged 50 or above from the 2016 Health and Retirement Study Venous Blood Study with measured DNAm. To assess heat exposure, we calculated daily heat index values at the census tract level using high-resolution meteorological data from gridMet and linked them to each respondent’s geocoded residential location. We considered both acute (on the day of blood collection) and chronic (1-year moving average) exposure windows. Multilevel regression models with natural cubic splines were used to assess the effects of heat exposure on epigenetic aging acceleration (i.e., Horvath, Hannum, PhenoAge, GrimAge, and DunedinPACE). Acute exposure to high heat index values (90th percentile compared to room temperature of 68F) was significantly associated with increased Horvath (0.77, p<.05), Hannum (0.73, p<.05), and PhenoAge (1.09, p<.001) acceleration. For long-term exposure, high heat index values were associated with increased PhenoAge (0.78, p<.05), GrimAge (0.52, p<.01), and DunedinPACE (0.02, p<.01). These findings provide insights into the biological underpinnings linking heat exposure to a broader spectrum of morbidity and mortality risks. Our study adds to the evidence highlighting the strong need to mitigate the adverse biological modifications induced by extreme heat.

